# Dr. Ernest P. Magruder

**Published:** 1915-05

**Authors:** 


**Dr. Ernest P. Magruder** of Washington, died of typhus while serving with the American Red Cross in Servia, early in April, aged 40. His is the second death from the same cause and in the same service in Servia, the first being that of Dr. Joseph F. Donnelly of Brooklyn.

